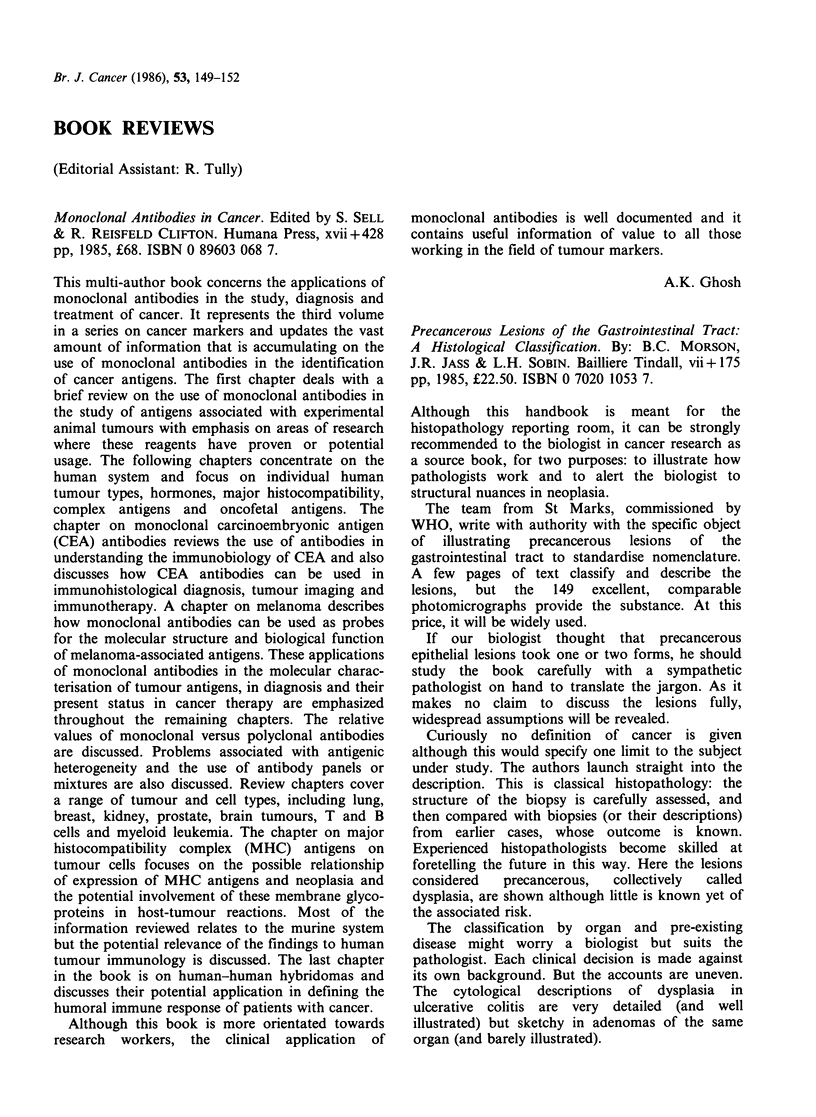# Monoclonal Antibodies in Cancer

**Published:** 1986-01

**Authors:** A.K. Ghosh


Br. J. Cancer (1986), 53, 149–152

### BOOK REVIEWS

(Editorial Assistant: R. Tully)

*Monoclonal Antibodies in Cancer*. Edited by S. SELL & R. REISFELD CLIFTON. Humana Press, xvii+428 pp, 1985, £68. ISBN 0 89603 068 7.

This multi-author book concerns the applications of monoclonal antibodies in the study, diagnosis and treatment of cancer. It represents the third volume in a series on cancer markers and updates the vast amount of information that is accumulating on the use of monoclonal antibodies in the identification of cancer antigens. The first chapter deals with a brief review on the use of monoclonal antibodies in the study of antigens associated with experimental animal tumours with emphasis on areas of research where these reagents have proven or potential usage. The following chapters concentrate on the human system and focus on individual human tumour types, hormones, major histocompatibility, complex antigens and oncofetal antigens. The chapter on monoclonal carcinoembryonic antigen (CEA) antibodies reviews the use of antibodies in understanding the immunobiology of CEA and also discusses how CEA antibodies can be used in immunohistological diagnosis, tumour imaging and immunotherapy. A chapter on melanoma describes how monoclonal antibodies can be used as probes for the molecular structure and biological function of melanoma-associated antigens. These applications of monoclonal antibodies in the molecular characterisation of tumour antigens, in diagnosis and their present status in cancer therapy are emphasized throughout the remaining chapters. The relative values of monoclonal versus polyclonal antibodies are discussed. Problems associated with antigenic heterogeneity and the use of antibody panels or mixtures are also discussed. Review chapters cover a range of tumour and cell types, including lung, breast, kidney, prostate, brain tumours, T and B cells and myeloid leukemia. The chapter on major histocompatibility complex (MHC) antigens on tumour cells focuses on the possible relationship of expression of MHC antigens and neoplasia and the potential involvement of these membrane glycoproteins in host-tumour reactions. Most of the information reviewed relates to the murine system but the potential relevance of the findings to human tumour immunology is discussed. The last chapter in the book is on human–human hybridomas and discusses their potential application in defining the humoral immune response of patients with cancer.

Although this book is more orientated towards research workers, the clinical application of monoclonal antibodies is well documented and it contains useful information of value to all those working in the field of tumour markers.

A.K. Ghosh